# Successful guidewire insertion into the gallbladder facilitated by a mixed-reality-based three-dimensional anatomical model

**DOI:** 10.1055/a-2687-6670

**Published:** 2025-09-04

**Authors:** Yuki Tanisaka, Shomei Ryozawa, Takao Itoi, Masafumi Mizuide, Akashi Fujita, Ryuichi Watanabe, Maki Sugimoto

**Affiliations:** 1 Department of Gastroenterology, Saitama Medical University International Medical Center, Saitama, Japan; 2 Department of Gastroenterology and Hepatology, Tokyo Medical University, Tokyo, Japan; 3 Innovation Lab, Okinaga Research Institute, Teikyo University, Tokyo, Japan

When endoscopic transpapillary gallbladder drainage (ETGBD) is required, it is challenging to advance the guidewire into the gallbladder when relying solely on two-dimensional imaging, such as computed tomography and cholangiography. Mixed reality is a technology that merges real and virtual environments in real time, creating an immersive and interactive experience 1 . By utilizing a dedicated head-mounted display and hand controllers (Meta Quest 3; Meta, Menlo Park, California, USA), clinicians can visualize computer-generated three-dimensional (3D) anatomical models, from any perspective ( Fig. 1 ). Several reports have demonstrated the feasibility of mixed-reality-based 3D anatomical models for pancreaticobiliary endoscopic procedures 2 3 4 . We present a case of successful guidewire insertion into the gallbladder facilitated by a mixed-reality-based 3D anatomical model in a patient with acute cholecystitis.

**Fig. 1:**
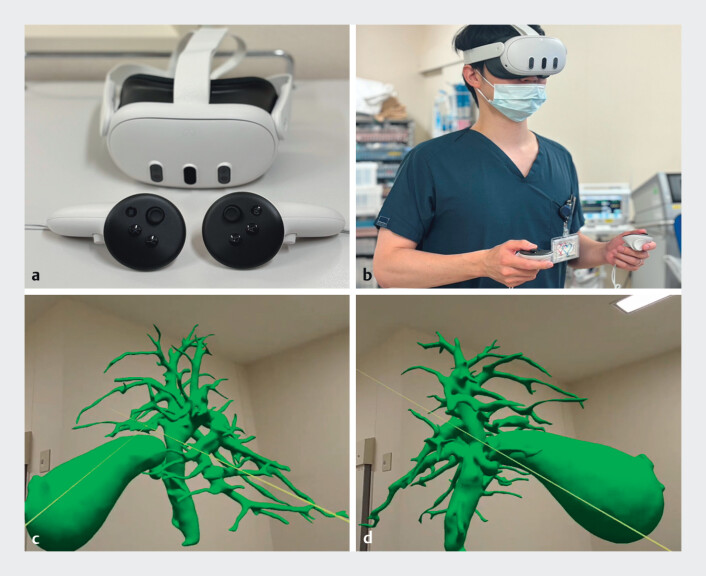
The mixed-reality-based three-dimensional (3D) anatomical model. **a** A dedicated head-mounted display and hand controllers. **b** A doctor wearing a head-mounted display and using hand controllers. **c** The computer-generated 3D anatomical model as seen through the head-mounted display. **d** The 3D anatomical model is rotatable.

A 76-year-old man who presented with acute cholecystitis was referred to us ( Fig. 2 ). Owing to his ineligibility for surgery and the presence of coagulopathy, ETGBD was selected 5 ( Video 1 ). A 3D anatomical model of the biliary system was created using magnetic resonance imaging with SYNAPSE VINCENT (Fujifilm Medical Co., Tokyo, Japan) and Holoeyes MD (Holoeyes Inc., Tokyo, Japan) ( Fig. 3 ). Before the procedure, endoscopists reviewed the patient-specific biliary anatomy, including the confluence of the common bile duct and cystic duct, using the mixed-reality-based 3D anatomical model ( Fig. 4 **a, b** ). During the procedure, the same model was referenced alongside the real-time fluoroscopic images ( Fig. 4 **c, d** ). The mixed-reality-based 3D anatomical model clearly visualized the anatomical configuration through rotation, which allowed the endoscopists to accurately identify the cystic duct and advance the guidewire into the gallbladder ( Fig. 5 **a** ). ETGBD was completed using a 7-Fr plastic stent ( Fig. 5 **b** ).

**Fig. 2:**
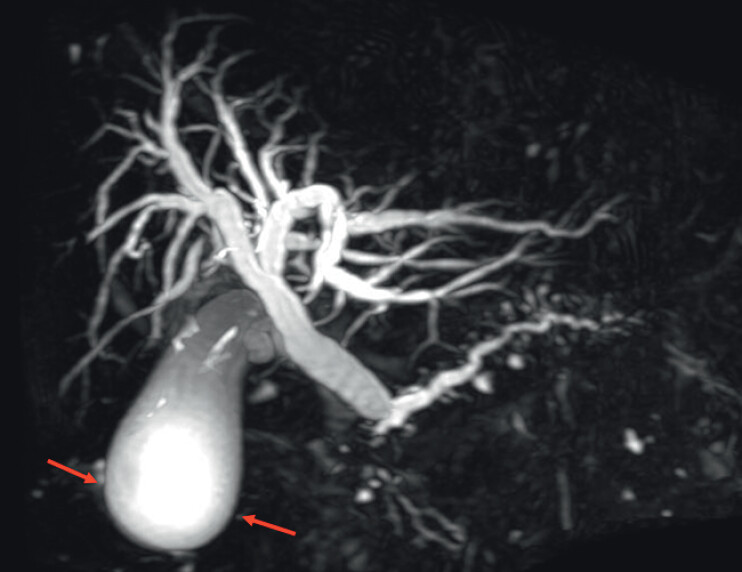
Magnetic resonance imaging revealed an enlarged gallbladder with wall thickening, indicating acute cholecystitis (red arrow).

Successful guidewire insertion into the gallbladder facilitated by the mixed-reality-based three-dimensional anatomical model.Video 1

**Fig. 3:**
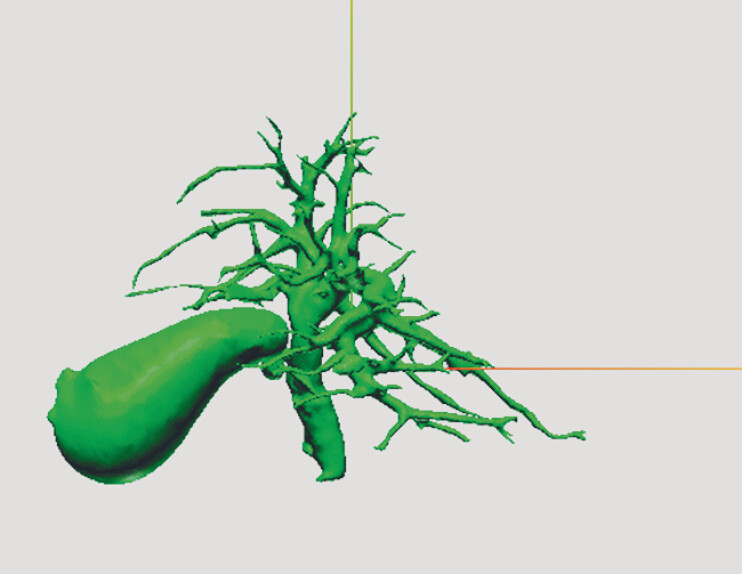
Magnetic resonance imaging was converted into a three-dimensional anatomical model.

**Fig. 4:**
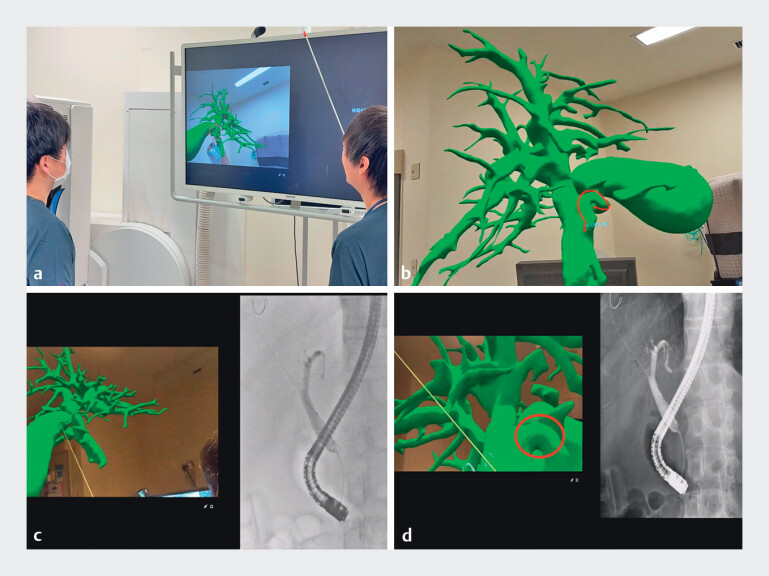
Use of the mixed-reality-based three-dimensional (3D) anatomical model. **a, b** Endoscopists using the model to review the patient-specific biliary anatomy, including the confluence of the common bile duct and cystic duct, before the procedure. **c, d** Endoscopists referencing the model alongside the real-time fluoroscopic images during the procedure.

**Fig. 5:**
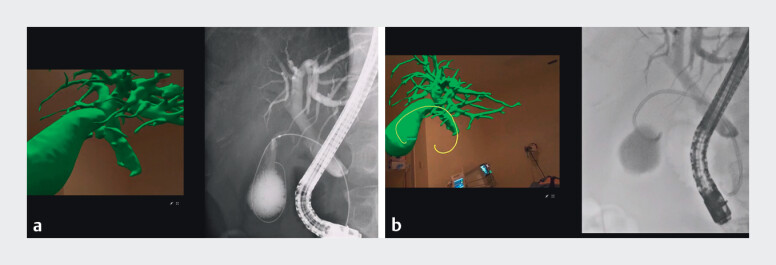
Guidewire insertion. **a** Successful guidewire insertion into the gallbladder. **b** Endoscopic transpapillary gallbladder drainage.

This case highlights the utility of a mixed-reality-based 3D anatomical model in both preoperative anatomical assessment and intraoperative navigation. The technology provided enhanced spatial understanding and real-time procedural support, suggesting its potential as an innovative educational and clinical tool for pancreaticobiliary endoscopists.

Endoscopy_UCTN_Code_TTT_1AR_2AB

## Notes

Endoscopy E-Videos https://eref.thieme.de/e-videos
*E-Videos* is an open access online section of the journal *Endoscopy* , reporting on interesting cases and new techniques in gastroenterological endoscopy. All papers include a high-quality video and are published with a Creative Commons CC-BY license. Endoscopy E-Videos qualify for HINARI discounts and waivers and eligibility is automatically checked during the submission process. We grant 100% waivers to articles whose corresponding authors are based in Group A countries and 50% waivers to those who are based in Group B countries as classified by Research4Life (see: https://www.research4life.org/access/eligibility/ ). This section has its own submission website at https://mc.manuscriptcentral.com/e-videos .
